# The genome sequence of the Gold Triangle, *Hypsopygia costalis* (Fabricius, 1775)

**DOI:** 10.12688/wellcomeopenres.18746.1

**Published:** 2023-01-11

**Authors:** Douglas Boyes, University of Oxford and Wytham Woods Genome Acquisition Lab, Wellcome Sanger Institute Tree of Life programme, Wellcome Sanger Institute Scientific Operations: DNA Pipelines collective, Tree of Life Core Informatics collective, James Hammond, Darwin Tree of Life Consortium

**Affiliations:** 1 UK Centre for Ecology and Hydrology, Wallingford, Oxfordshire, UK; 2 Department of Biology, University of Oxford, Oxford, Oxfordshire, UK

**Keywords:** Hypsopygia costalis, Gold Triangle, genome sequence, chromosomal, Lepidoptera

## Abstract

We present a genome assembly from an individual male *Hypsopygia costalis* (the Gold Triangle; Arthropoda; Insecta; Lepidoptera; Pyralidae). The genome sequence is 818 megabases in span. Most of the assembly is scaffolded into 31 chromosomal pseudomolecules with the Z sex chromosome assembled. The mitochondrial genome has also been assembled and is 15.3 kilobases in length. Gene annotation of this assembly on Ensembl identified 19,248 protein coding genes.

## Species taxonomy

Eukaryota; Metazoa; Ecdysozoa; Arthropoda; Hexapoda; Insecta; Pterygota; Neoptera; Endopterygota; Lepidoptera; Glossata; Ditrysia; Pyraloidea; Pyralidae; Pyralinae; *Hypsopygia*; *Hypsopygia costalis* (Fabricius, 1775) (NCBI:txid1101110).

## Background

*Hypsopygia costalis* (Fabricius, 1775) is a moth in the Pyralidae family, known as the Gold Triangle in the British Isles, and the Clover Hayworm in North America. Its British and Irish common name derives from the characteristic golden yellow triangular markings formed where the narrow fasciae broaden as they meet the costa. Within the British Isles, the moth is most often encountered in England and Wales, becoming scarcer as it moves north towards the extremity of its range in the Scottish Borders ( Cubitt, 2021; Parsons & Davis, 2018). The species is apparently absent from Ireland, with only three records that may constitute accidental imports ( Walsh *et al.*, 2009). Globally, the moth occurs in Europe and eastern North America ( GBIF Secretariat, 2021).

The larva feeds on dried vegetation, most notably hay made from clover or alfalfa, of which it can be a serious pest, thus earning it its North American common name ( Goater *et al.*, 1986; Parsons & Davis, 2018; Swenk, 1908). The species is also thought to feed on thatch and has even been reported feeding on vegetable matter within a squirrel’s drey ( Goater *et al.*, 1986). Pupation occurs within an oval cocoon in the feeding locale ( Parsons & Davis, 2018). The adult moth measures 18–22 mm in wingspan and is on the wing from July to November ( Goater *et al.*, 1986; Parsons & Davis, 2018). It is nocturnal, resting by day in thatch and hedgerows, or in the case of a hay infestation, can be found resting on the walls of barns ( Goater *et al.*, 1986; Swenk, 1908). The adult is attracted to light, and has also been reported at sugar ( Swenk, 1908).

The genome of *H. costalis* was sequenced as part of the Darwin Tree of Life Project, a collaborative effort to sequence all named eukaryotic species in the Atlantic Archipelago of Britain and Ireland. Here we present a chromosomally complete genome sequence for *H. costalis*, based on one male specimen from Wytham Woods, Berkshire, UK.

## Genome sequence report

**Figure 1.**
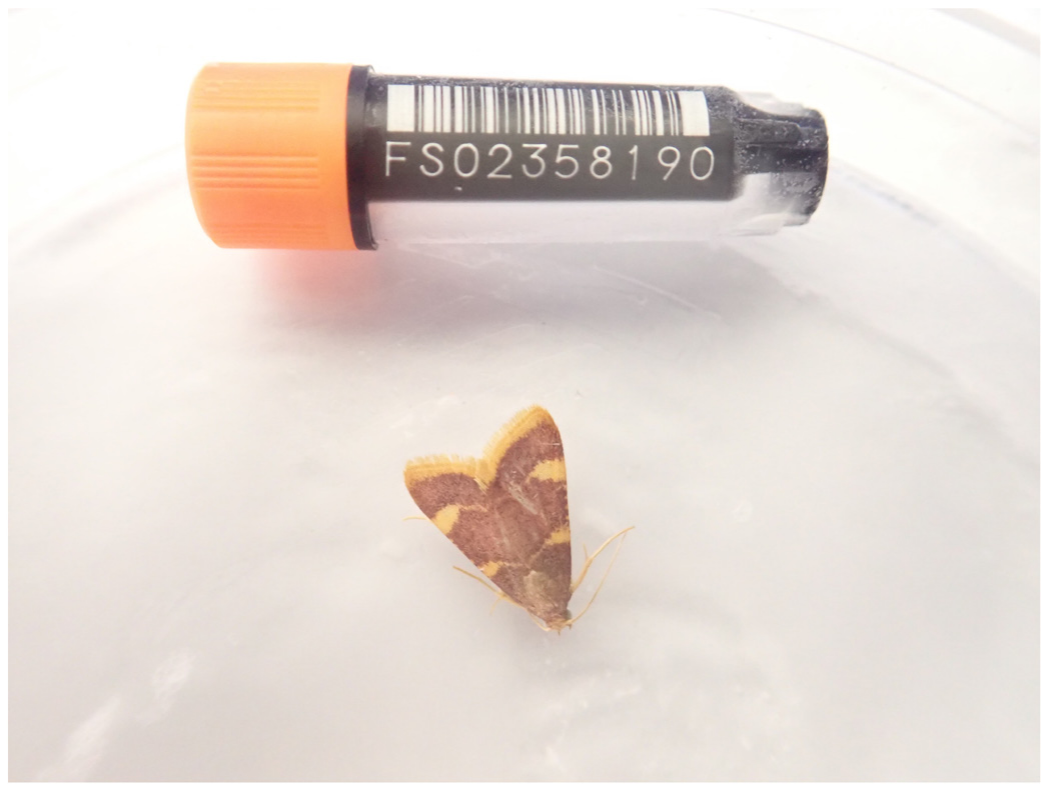
Photograph of the *Hypsopygia costalis* (ilHypCost1) specimen used for genome sequencing.

The genome was sequenced from one male *H. costalis* ( Figure 1) collected from Wytham Woods, Berkshire, UK (latitude 51.77, longitude –1.34). A total of 50-fold coverage in Pacific Biosciences single-molecule HiFi long reads and 38-fold coverage in 10X Genomics read clouds was generated. Primary assembly contigs were scaffolded with chromosome conformation Hi-C data. Manual assembly curation corrected seven missing or mis-joins and removed one haplotypic duplication, reducing the scaffold number by 7.69%.

**Figure 2.**
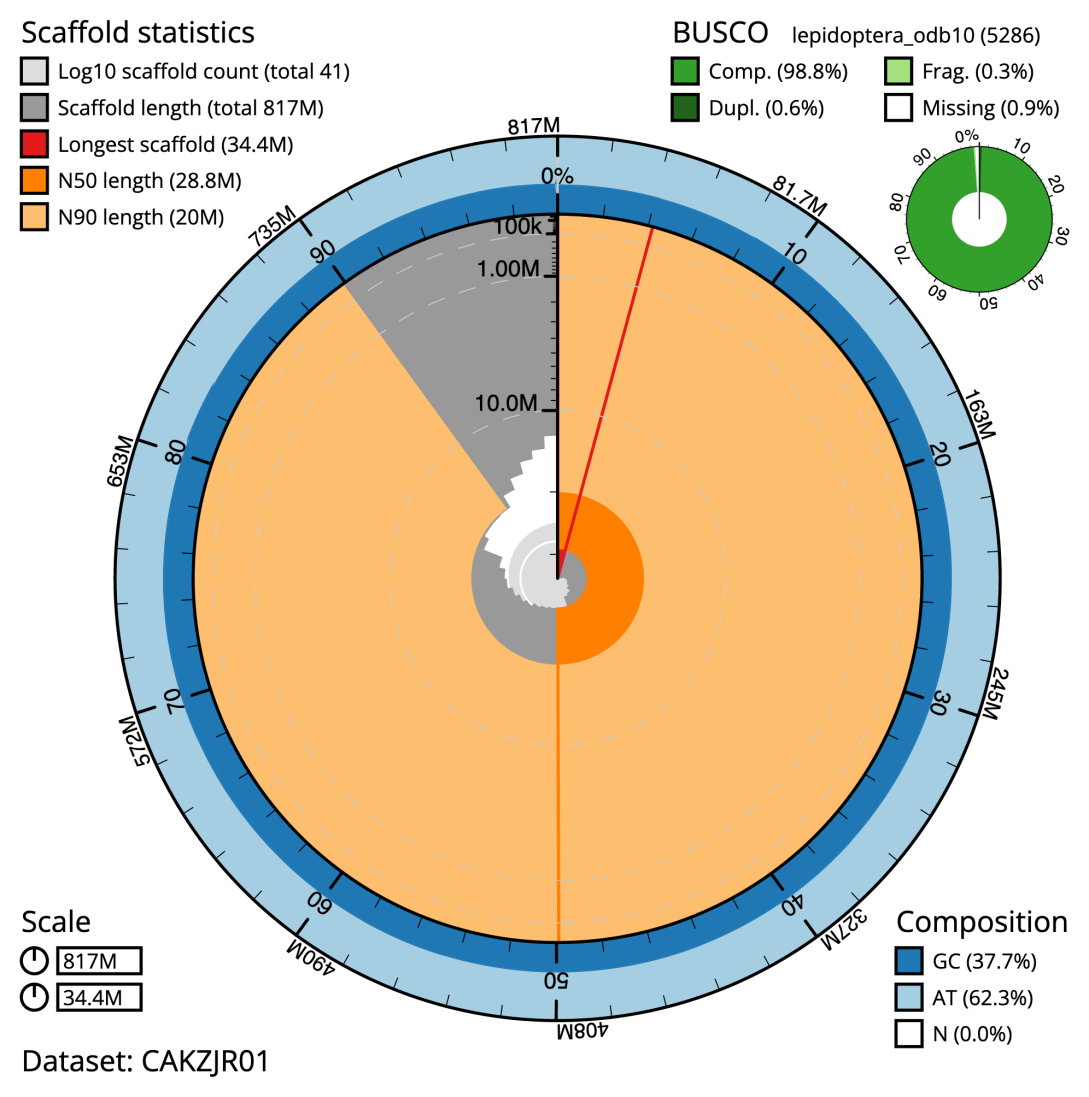
Genome assembly of *Hypsopygia costalis*, ilHypCost1.2: metrics. The BlobToolKit Snailplot shows N50 metrics and BUSCO gene completeness. The main plot is divided into 1,000 size-ordered bins around the circumference with each bin representing 0.1% of the 816,870,447 bp assembly. The distribution of scaffold lengths is shown in dark grey with the plot radius scaled to the longest sequence present in the assembly (34,377,200 bp, shown in red). Orange and pale-orange arcs show the N50 and N90 sequence lengths (28,834,930 and 20,016,790 bp), respectively. The pale grey spiral shows the cumulative scaffold count on a log scale with white scale lines showing successive orders of magnitude. The blue and pale-blue area around the outside of the plot shows the distribution of GC, AT and N percentages in the same bins as the inner plot. A summary of complete, fragmented, duplicated and missing BUSCO genes in the lepidoptera_odb10 set is shown in the top right. An interactive version of this figure is available at https://blobtoolkit.genomehubs.org/view/Hypsopygia%20costalis/dataset/CAKZJR01/snail.

The final assembly has a total length of 817.7 Mb in 48 sequence scaffolds with a scaffold N50 of 28.9 Mb ( Table 1). Most (99.91%) of the assembly sequence was assigned to 31 chromosomal-level scaffolds, representing 30 autosomes and the Z sex chromosome. Chromosome-scale scaffolds confirmed by the Hi-C data are named in order of size ( Figure 2– Figure 5; Table 2). The assembly has a BUSCO v5.3.2 ( Manni *et al.*, 2021) completeness of 98.8% (single 98.2%, duplicated 0.6%) using the OrthoDB v10 lepidoptera reference set. While not fully phased, the assembly deposited is of one haplotype. Contigs corresponding to the second haplotype have also been deposited.

**Table 1.** Genome data for *Hypsopygia costalis*, ilHypCost1.2.

Project accession data		
Assembly identifier	ilHypCost1.2.	
Species	*Hypsopygia costalis*	
Specimen	ilHypCost1	
NCBI taxonomy ID	1101110	
BioProject	PRJEB51267	
BioSample ID	SAMEA7701325	
Isolate information	male: ilHypCost1 (PacBio, 10X sequencing), female: ilHypCost2 (Hi-C)	
Assembly metrics *		*Benchmark*
Consensus quality (QV)	58.2	*≥ 50*
*k*-mer completeness	100%	*≥ 95%*
BUSCO **	C:98.8%[S:98.2%,D:0.6%], F:0.3%,M:0.9%,n:5,286	*C ≥ 95%*
Percentage of assembly mapped to chromosomes	99.91%	*≥ 95%*
Sex chromosomes	Z chromosome	*localised homologous pairs*
Organelles	Mitochondrial genome assembled	*complete single alleles*
Raw data accessions		
PacificBiosciences SEQUEL II	ERR9127941, ERR9468770	
10X Genomics Illumina	ERR9123818–ERR9123821	
Hi-C Illumina	ERR9123822	
Genome assembly		
Assembly accession	GCA_937001555.2	
*Accession of alternate* *haplotype*	GCA_937001695.1	
Span (Mb)	817.7	
Number of contigs	75	
Contig N50 length (Mb)	2.7	
Number of scaffolds	48	
Scaffold N50 length (Mb)	28.9	
Longest scaffold (Mb)	34.4	
Genome annotation		
Number of protein-coding genes	19,248	
Number of gene transcripts	19,419	

* Assembly metric benchmarks are adapted from column VGP-2020 of “Table 1: Proposed standards and metrics for defining genome assembly quality” from ( Rhie *et al.*, 2021).

** BUSCO scores based on the lepidoptera_odb10 BUSCO set using v5.3.2. C = complete [S = single copy, D = duplicated], F = fragmented, M = missing, n = number of orthologues in comparison. A full set of BUSCO scores is available at https://blobtoolkit.genomehubs.org/view/Hypsopygia%20costalis/dataset/CAKZJR01/busco.

**Figure 3.**
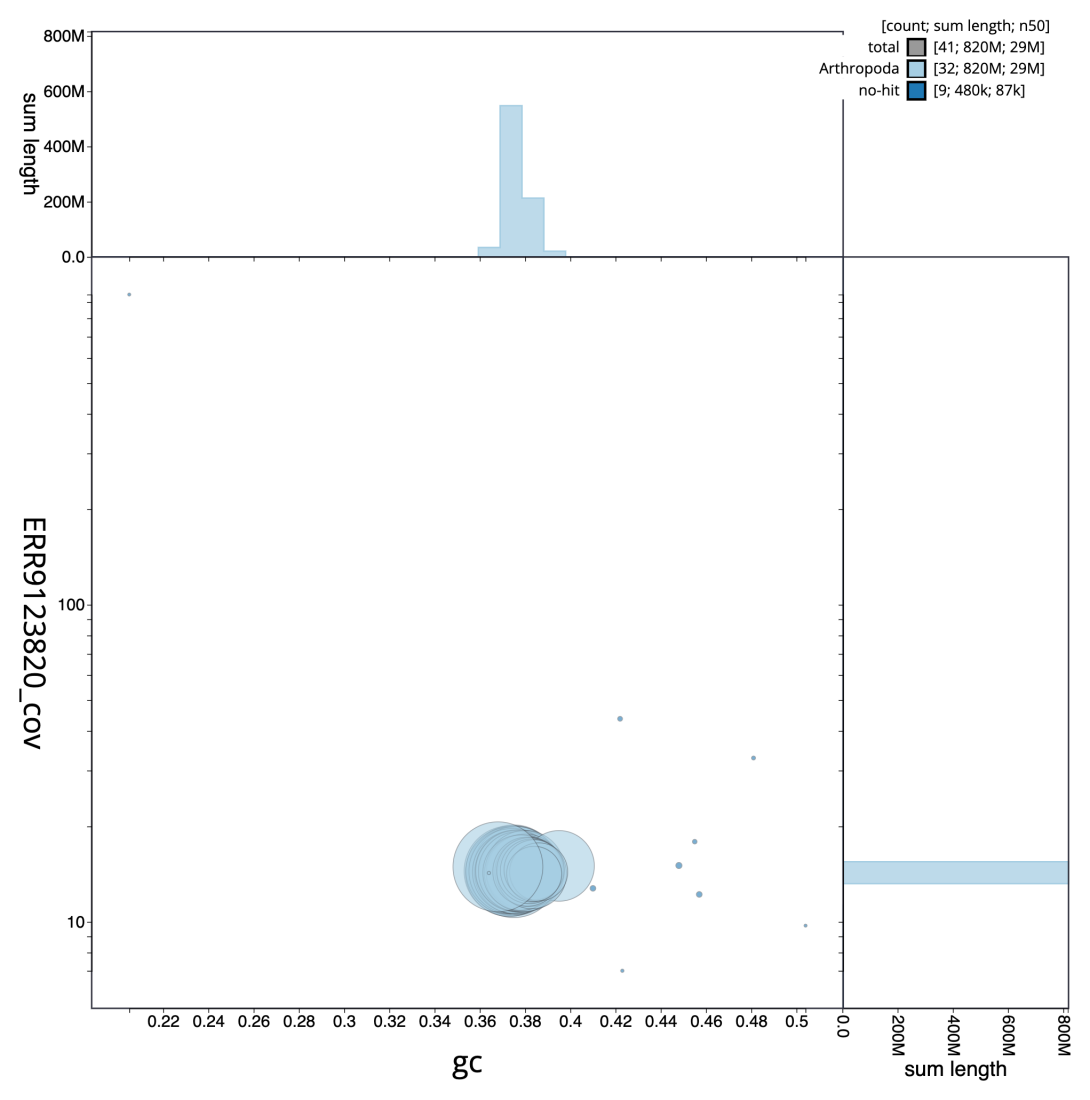
Genome assembly of *Hypsopygia costalis*, ilHypCost1.2: GC coverage. BlobToolKit GC-coverage plot. Scaffolds are coloured by phylum. Circles are sized in proportion to scaffold length. Histograms show the distribution of scaffold length sum along each axis. An interactive version of this figure is available at https://blobtoolkit.genomehubs.org/view/Hypsopygia%20costalis/dataset/CAKZJR01/blob.

**Figure 4.**
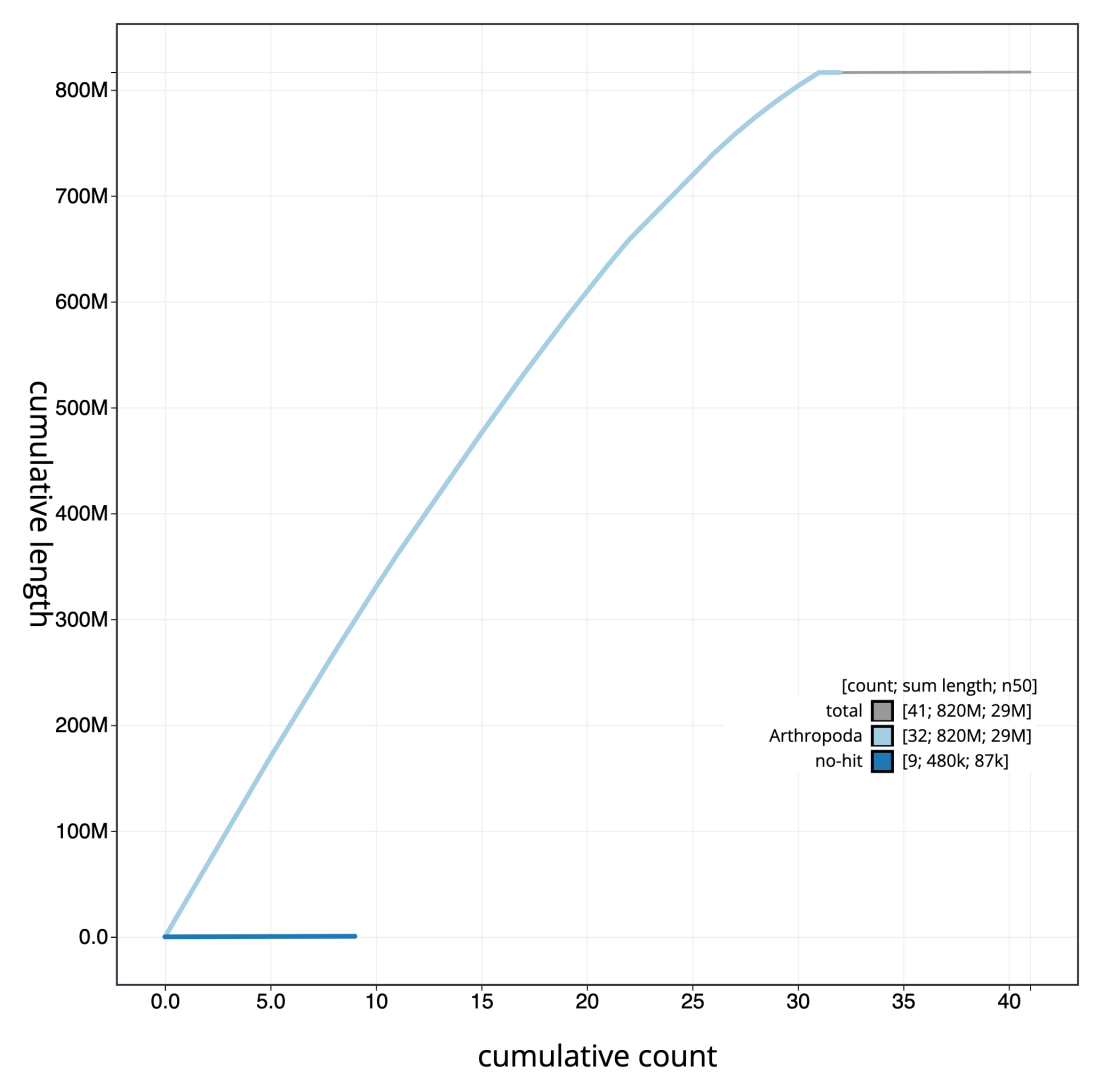
Genome assembly of *Hypsopygia costalis*, ilHypCost1.2: cumulative sequence. BlobToolKit cumulative sequence plot. The grey line shows cumulative length for all scaffolds. Coloured lines show cumulative lengths of scaffolds assigned to each phylum using the buscogenes taxrule. An interactive version of this figure is available at https://blobtoolkit.genomehubs.org/view/Hypsopygia%20costalis/dataset/CAKZJR01/cumulative.

**Figure 5.**
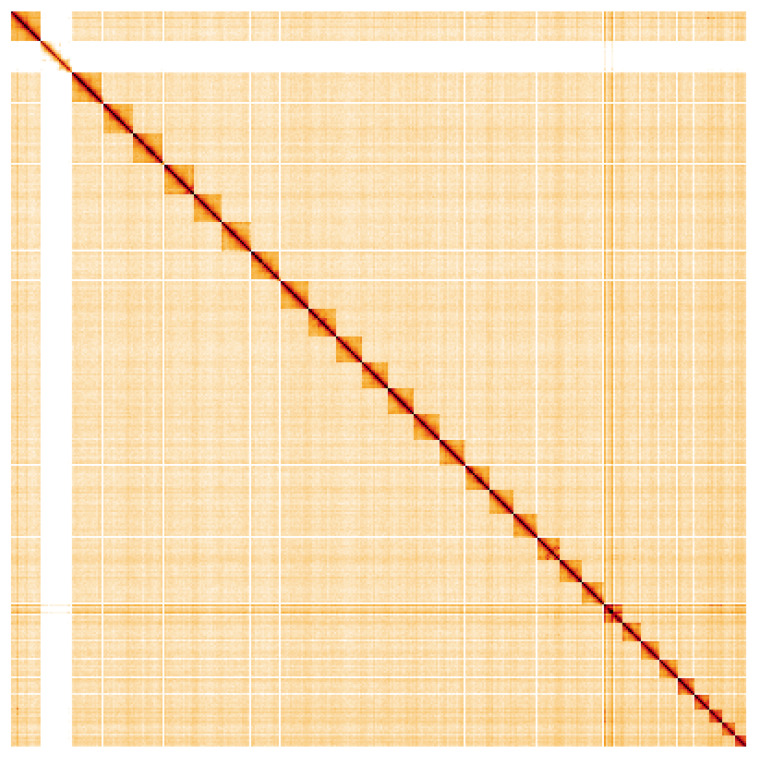
Genome assembly of *Hypsopygia costalis*, ilHypCost1.2: Hi-C contact map. Hi-C contact map of the ilHypCost1.2 assembly, visualised using HiGlass. The female specimen lHypCost2 was used to generate the Hi-C library. Chromosomes are shown in order of size from left to right and top to bottom. An interactive version of this figure may be viewed at https://genome-note-higlass.tol.sanger.ac.uk/l/?d=TGGC-QK7QQujf69Q2nhz8g.

**Table 2.** Chromosomal pseudomolecules in the genome assembly of *Hypsopygia costalis*, ilHypCost1.

INSDC accession	Chromosome	Size (Mb)	GC content (%)
OW443325.1	1	34.38	37
OW443327.1	2	34.08	37
OW443328.1	3	33.84	37.5
OW443329.1	4	33.60	37
OW443330.1	5	32.82	37.5
OW443331.1	6	32.43	37
OW443332.1	7	32.22	37
OW443333.1	8	31.76	37
OW443334.1	9	30.97	37.5
OW443335.1	10	30.60	37
OW443336.1	11	28.89	37.5
OW443337.1	12	28.83	37.5
OW443338.1	13	28.79	37.5
OW443339.1	14	28.49	37.5
OW443340.1	15	28.07	37.5
OW443341.1	16	27.42	37.5
OW443342.1	17	26.85	37.5
OW443343.1	18	26.11	37.5
OW443344.1	19	25.31	37.5
OW443345.1	20	24.95	37.5
OW443346.1	21	23.97	37.5
OW443347.1	22	20.93	39.5
OW443348.1	23	20.17	38
OW443349.1	24	20.14	38
OW443350.1	25	20.02	38
OW443351.1	26	18.16	38
OW443352.1	27	16.62	38
OW443353.1	28	15.10	38
OW443354.1	29	14.08	38.5
OW443355.1	30	12.61	38
OW443326.1	Z	34.14	36.5
OW443356.1	MT	0.02	20.5

## Genome annotation report

The GCA_937001555.1 genome assembly was annotated using the Ensembl rapid annotation pipeline ( Table 1; https://rapid.ensembl.org/Hypsopygia_costalis_GCA_937001555.1/). The resulting annotation includes 19,419 transcripts from 19,248 protein-coding genes.

## Methods

### Sample acquisition and nucleic acid extraction

Two *H. costalis* (ilHypCost1 and ilHypCost2) specimens were collected in Wytham Woods, Berkshire, UK (latitude 51.77, longitude –1.34) using a light trap. The specimens were collected and identified by Douglas Boyes (University of Oxford) and snap-frozen on dry ice.

DNA was extracted at the Tree of Life laboratory, Wellcome Sanger Institute (WSI). The ilHypCost1 sample was weighed and dissected on dry ice. Whole organism tissue was disrupted using a Nippi Powermasher fitted with a BioMasher pestle. High molecular weight (HMW) DNA was extracted using the Qiagen MagAttract HMW DNA extraction kit. Low molecular weight DNA was removed from a 20 ng aliquot of extracted DNA using 0.8X AMpure XP purification kit prior to 10X Chromium sequencing; a minimum of 50 ng DNA was submitted for 10X sequencing. HMW DNA was sheared into an average fragment size of 12–20 kb in a Megaruptor 3 system with speed setting 30. Sheared DNA was purified by solid-phase reversible immobilisation using AMPure PB beads with a 1.8X ratio of beads to sample to remove the shorter fragments and concentrate the DNA sample. The concentration of the sheared and purified DNA was assessed using a Nanodrop spectrophotometer and Qubit Fluorometer and Qubit dsDNA High Sensitivity Assay kit. Fragment size distribution was evaluated by running the sample on the FemtoPulse system.

### Sequencing

Pacific Biosciences HiFi circular consensus and 10X Genomics read cloud DNA sequencing libraries were constructed according to the manufacturers’ instructions. DNA sequencing was performed by the Scientific Operations core at the WSI on Pacific Biosciences SEQUEL II (HiFi) and Illumina NovaSeq 6000 (10X) instruments. Hi-C data were also generated from ilHypCost2 using the Arima v2 kit and sequenced on the Illumina NovaSeq 6000 instrument.

### Genome assembly

Assembly was carried out with Hifiasm ( Cheng *et al.*, 2021) and haplotypic duplication was identified and removed with purge_dups ( Guan *et al.*, 2020). One round of polishing was performed by aligning 10X Genomics read data to the assembly with Long Ranger ALIGN, calling variants with freebayes ( Garrison & Marth, 2012). The assembly was then scaffolded with Hi-C data ( Rao *et al.*, 2014) using YaHS ( Zhou *et al.*, 2022). The assembly was checked for contamination as described previously ( Howe *et al.*, 2021). Manual curation was performed using HiGlass ( Kerpedjiev *et al.*, 2018) and Pretext ( Harry, 2022). The mitochondrial genome was assembled using MitoHiFi ( Uliano-Silva *et al.*, 2021), which performed annotation using MitoFinder ( Allio *et al.*, 2020). The genome was analysed and BUSCO scores generated within the BlobToolKit environment ( Challis *et al.*, 2020). Table 3 contains a list of all software tool versions used, where relevant.

**Table 3.** Software tools and versions used.

Software tool	Version	Source
BlobToolKit	3.4.0	Challis *et al.*, 2020
freebayes	1.3.1-17-gaa2ace8	Garrison & Marth, 2012
Hifiasm	0.16.1-r375	Cheng *et al.*, 2021
HiGlass	1.11.6	Kerpedjiev *et al.*, 2018
Long Ranger ALIGN	2.2.2	https://support.10xgenomics.com/genome-exome/software/ pipelines/latest/advanced/other-pipelines
MitoHiFi	2	Uliano-Silva *et al.*, 2021
PretextView	0.2	Harry, 2022
purge_dups	1.2.3	Guan *et al.*, 2020
YaHS	yahs-1.1.91eebc2	Zhou *et al.*, 2022

### Genome annotation

The BRAKER2 pipeline ( Brůna *et al.*, 2021) was used to annotate the *H. costalis* genome assembly (GCA_937001555.1) in Ensembl Rapid Release. BRAKER2 performs automatic gene annotation as a draft annotation without transcriptomic data.

### Ethics/compliance issues

The materials that have contributed to this genome note have been supplied by a Darwin Tree of Life Partner. The submission of materials by a Darwin Tree of Life Partner is subject to the Darwin Tree of Life Project Sampling Code of Practice. By agreeing with and signing up to the Sampling Code of Practice, the Darwin Tree of Life Partner agrees they will meet the legal and ethical requirements and standards set out within this document in respect of all samples acquired for, and supplied to, the Darwin Tree of Life Project. Each transfer of samples is further undertaken according to a Research Collaboration Agreement or Material Transfer Agreement entered into by the Darwin Tree of Life Partner, Genome Research Limited (operating as the Wellcome Sanger Institute), and in some circumstances other Darwin Tree of Life collaborators.

## Data Availability

European Nucleotide Archive: *Hypsopygia costalis* (gold triangle). Accession number PRJEB51267; https://identifiers.org/ena.embl/PRJEB51267. The genome sequence is released openly for reuse. The *Hypsopygia costalis* genome sequencing initiative is part of the Darwin Tree of Life (DToL) project. All raw sequence data and the assembly have been deposited in INSDC databases. Raw data and assembly accession identifiers are reported in Table 1.

## Author information

Members of the University of Oxford and Wytham Woods Genome Acquisition Lab are listed here: https://doi.org/10.5281/zenodo.4789928.

Members of the Darwin Tree of Life Barcoding collective are listed here: https://doi.org/10.5281/zenodo.4893703.

Members of the Wellcome Sanger Institute Tree of Life programme are listed here: https://doi.org/10.5281/zenodo.4783585.

Members of Wellcome Sanger Institute Scientific Operations: DNA Pipelines collective are listed here: https://doi.org/10.5281/zenodo.4790455.

Members of the Tree of Life Core Informatics collective are listed here: https://doi.org/10.5281/zenodo.5013541.

Members of the Darwin Tree of Life Consortium are listed here: https://doi.org/10.5281/zenodo.4783558.
