# Supplementary material for: The genome sequence of the Gold Triangle, Hypsopygia costalis (Fabricius, 1775)
Source: Wellcome Open Res. 2023 Jan 11;8:15. [Version 1] doi: 10.12688/wellcomeopenres.18746.1 (PMC10687387; doi:10.12688/wellcomeopenres.18746.1)
Supplement: Peer review history

# Reviewer report: Reviewer response for version 1

**DOI:** 10.21956/wellcomeopenres.20785.r62218

**Published:** 2023-11-29

**Authors:** Jeffrey Vedanayagam

**Affiliations:** 1 Department of Neuroscience, Developmental and Regenerative Biology, The University of Texas at San Antonio, San Antonio, Texas, USA

The methods are clearly described, and the protocols used are current and technically sound. Although the data is not phased, the authors have acknowledged this and provided data from two haplotypes. The figures are clear and easy to interpret, and informative. The data is publicly available and the authors have deposited it to the EMBL biobank.

Are sufficient details of methods and materials provided to allow replication by others?

Yes

Is the rationale for creating the dataset(s) clearly described?

Yes

Are the datasets clearly presented in a useable and accessible format?

Yes

Are the protocols appropriate and is the work technically sound?

Yes

Reviewer Expertise:

Genomics, evolutionary biology, bioinformatics.

I confirm that I have read this submission and believe that I have an appropriate level of expertise to confirm that it is of an acceptable scientific standard.

# Reviewer report: Reviewer response for version 1

**DOI:** 10.21956/wellcomeopenres.20785.r62215

**Published:** 2023-11-29

**Authors:** Fathiya Mbarak Khamis, Inusa Jacob Ajene

**Affiliations:** 1 Plant Health Theme, International Centre for Insect Physiology and Ecology, Nairobi, Nairobi County, Kenya; 2 International Centre of Insect Physiology and Ecology, Nairobi, Kenya

The data note titled “The genome sequence of the Gold Triangle, *Hypsopygia costalis *(Fabricius, 1775)” presents the genome assembly of the Gold Triangle moth *Hypsopygia costalis *(Lepidoptera; Pyralidae). This pest has significant agricultural implications as it has been found to be injurious on clover hay and other types of hay, causing production losses as affected hay becomes contaminated with the caterpillar webbings and frass, thus, making it unsuitable for use as feed. Furthermore, the main control strategy for this pest requires the burning of the affected hay, which leads to further loss. The potential for invasion of this moth is also a cause for concern to production regions outside Europe where the moth is yet to be reported. Therefore, this is a very good paper with impact as a potential resource for future genomic studies, including population genetics, phylogeography, invasive potential, and identification of potential biological management options of the Gold Triangle moth. The note is thorough, describing the background of the insect, the genome assembly and annotation. The methodology is sound, and the results present a comprehensive view of the study genome.

 My minor suggestions are:

 The authors should revise the statement in the introduction, which reads, “ *The adult is attracted to light, and has also been reported at sugar*”. Is the moth attracted to sugar, or was it found in sugar? This is a bit confusing either way, the statement should be rephrased properly.

Are sufficient details of methods and materials provided to allow replication by others?

Yes

Is the rationale for creating the dataset(s) clearly described?

Yes

Are the datasets clearly presented in a useable and accessible format?

Yes

Are the protocols appropriate and is the work technically sound?

Yes

Reviewer Expertise:

Molecular biology

We confirm that we have read this submission and believe that we have an appropriate level of expertise to confirm that it is of an acceptable scientific standard.
